# Construction of nursing-sensitive quality indicators for pregnancy-associated venous thromboembolism using the Delphi method

**DOI:** 10.1186/s12912-026-04517-y

**Published:** 2026-03-07

**Authors:** Wei Yue, Yu-Yi Gao, Chen Qin, Shan-Xia Chen, Min Yang, Xia-Xia Lin, De-Mei Lu

**Affiliations:** 1 Department of Obstetrics, the Affiliated Foshan Women and Children Hospital, Guangdong Medical University, No.20, Huayang South Road, Shunde District, Guangdong Foshan, China; 2 Health Management&Biotechnology School, Guangdong Food and Drug Vocational College, Guangzhou, Guangdong China; 3 Foshan Third People’s Hospital, Foshan, Guangdong China

**Keywords:** Pregnancy-associated venous thromboembolism, Quality indicators, Delphi method, Analytic hierarchy process, Nursing management

## Abstract

**Background:**

There is an increasing trend of Pregnancy-associated Venous Thromboembolism (PA-VTE) over the years in China due to postponement of childbearing age, obesity, and the increasing incidence of pregnancy complications, thereby posing a substantial threat to maternal safety. However, there is a lack of a unified standard indicator system for women with PA-VTE. This study aimed to develop applicable nursing-sensitive quality indicators for women with PA-VTE.

**Methods:**

Potential nursing-sensitive quality indicators were initially constructed based on literature review and research group discussion. A Delphi panel of eighteen experts (obstetric nurses, evidence-based nurses, nurse managers) participated in two rounds consultation.

**Results:**

The constructed nursing-sensitive quality indicators for the women with PA-VTE included 3 primary indicators, 9 secondary indicators and 24 tertiary indicators. The response rate of the two rounds of expert survey questionnaires was 100%, the expert authority coefficient values of 0.89, and Kendall coordination coefficient *W* values ranging from 0.093 to 0.136.

**Conclusions:**

The final set of NSQIs, structured according to the Donabedian model, provides a quantifiable framework to guide clinical nursing practice and quality management for women with PA-VTE. Future work should focus on implementing these indicators in different hospital settings, evaluating their feasibility and reliability in routine data collection, and refining the indicator set based on empirical performance and patient outcomes.

**Clinical trial number:**

Not applicable.

**Supplementary information:**

The online version contains supplementary material available at 10.1186/s12912-026-04517-y.

## Introduction

Pregnancy-associated Venous thromboembolism (PA-VTE) refers to a specific type of venous thromboembolism (VTE) that arises from the interplay of obstetric and non-obstetric factors, influenced by the unique physiological conditions present during pregnancy and the puerperium [1]. VTE, including deep venous thrombosis (DVT) and pulmonary embolism (PE), is a major cause of morbidity and mortality during pregnancy and the postpartum period [2, 3]. DVT represents the majority of pregnancy-associated VTE, but PE is often of greater concern as it leads to the majority of VTE-related morbidity and contributes to 10%-15% of pregnancy associated maternal deaths in high-income countries [4, 5]. According to reports, 78,000 pregnant women die from PA-VTE worldwide annually, making it the second leading cause of maternal mortality in developed countries [6]. A population-based epidemiological study spanning from 2000 to 2018 revealed that the incidence of PA-VTE in the United States was approximately 0.066% [7], and in China, it was 0.13% in 2023 [8]. However, with the postponement of childbearing age, obesity, and the increasing incidence of pregnancy complications, there is an increasing trend of PA-VTE over the years in China [9].

PA-VTE reduces maternal life quality and is associated with substantial health care costs [10]. Thus, improving PA-VTE’ nursing quality evaluation system, improving the effectiveness of nurse managers, and ensuring the safety of the nursing care provided are the key points of nursing management. Proposed by Podgomey [11], nursing sensitive quality indicators (NSQIs) play a crucial role in measuring the specific quality of care inherent in nursing practice [12, 13]. NSQIs are defined as the procedures and outcomes of patient-oriented nursing services, they are assessed by leveraging nursing data and quantitative evaluation methods, while also monitoring a range of functional qualities—such as nursing management and clinical practice—that have a direct bearing on patient outcomes [14–16]. NSQIs are quantitative metrics for measuring nursing care quality, as well as important tools for nursing quality management in hospitals [15].

In 1966, Avedis Donabedian put forward the theoretical framework of a three-dimensional quality structure, which divides medical quality into three core dimensions: structure, process and outcome. This model has since served as the primary theoretical and framework basis for developing NSQIs [17]. In 2016, the nursing center of China’s Hospital Management Institute proposed 13 NSQIs, none of which are related to PA-VTE [18]. To the best of our knowledge, studies worldwide focusing on the construction of NSQIs for VTE are still insufficient in both quantity and scope. Most of the available studies has targeted adult hospitalized patients [19, 20] and those with gynecological malignant tumors [21]. By contrast, the development of specialized nursing quality evaluation index systems for PA-VTE has not been systematically explored, leaving a critical gap in this field. Therefore, based on the Donabedian structure-process-result theory model, this study intends to construct scientific and reasonable NSQIs to provide standardized guidance tools for providing nursing care for women with PA-VTE.

## Methods

### Design

**Fig. 1:**
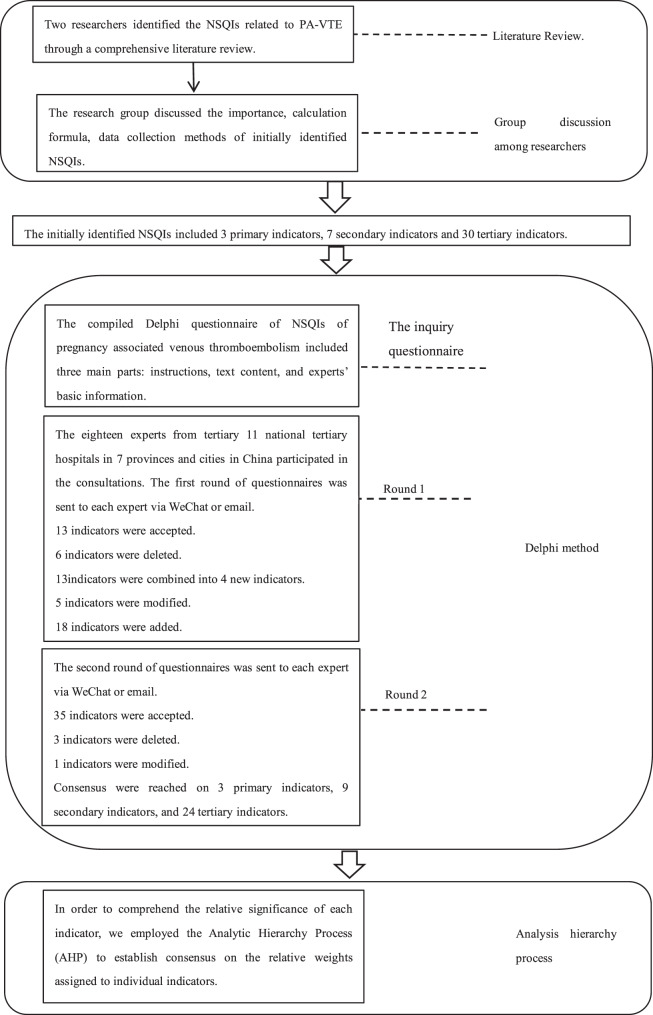
Flowchart of the study

The study consisted of four phases, Fig. 1 depicts the fundamental process of identifying the indicators in this study.

### Literature source and retrieval method

The research group consisted of three obstetric head nurses, two VTE specialist obstetric nurses, and two master’s degree obstetric nurses with a background in evidence-based nursing. Based on the “6S” evidence source gold tower model [22], two master’s obstetric nurses searched the BMJ BestPractice, UpToDate, Cochrane Library, Joanna Briggs Institute, National Guideline Clearinghouse, National Institute for Health and Care Excellence, Scottish Intercollegiate Guidelines Network, Registered Nurses Association of Ontario, Web of Science, PubMed, EBSCO, Embase, Medline, Cochrane library, CBM, CNKI, Wanfang, VIP databases for eligible studies published from inception to June 2024 using the key words “maternity”, “pregnancy”, “perinatal care”, “obstetrics”, “postpartum period”, “cesarean section”, “deep venous thrombosis”, “vein thrombosis”, “venous thromboembolism”, “pulmonary thromboembolism”, “pulmonary embolism”, “quality indicators”, “sensitive indicators”, “nursing indicator”, “assessment index”, “index of nursing”. Two researchers independently identified the NSQIs associated with PA-VTE. Subsequently, they collaborated to consolidate the acquired results and sought input from a third researcher to address any disparities.

### Construction of the entry pool

Two researchers conducted a thorough review of the literature, extracting information and acquiring the connotation, calculation formula, and data collection method for NSQIs from the Practical Handbook of Care-Sensitive Quality Indicators in China [23]. Based on the extracted contents and clinical nursing experience, the remaining members proposed suggestions. All group members discussed the classification, definition and calculation formula of NSQIs based on the Donabedian’s theoretical model [17]. Finally, we developed entry pool of NSQIs related to PA-VTE, including three primary indicators (structural indicators, process indicators, and outcome indicators), seven secondary indicators (management system, equipment configuration, teaching and training, nursing evaluation, nursing intervention, maternal outcome, and nursing efficacy), and 29 tertiary indicators (implementation rate of regular training for VTE prevention and treatment team, qualification rate of VTE equipment management, usage rate of gradient pressure elastic socks,usage rate of intermittent inflation and pressurization device, etc.).

### Construction of the Delphi questionnaire

The compiled Delphi questionnaire had three main parts: the first part described the questionnaire, including research background, purpose, significance, and instructions for completion. The second part focused on indicator content, providing an overview of sensitive indicators such as definitions and calculation formulas. Experts utilized the Likert 5-point scale to evaluate the importance of indicator content, the rationality of calculation formulas, and the feasibility of data collection. Ratings ranged from “very unimportant/very unreasonable/very unfeasible” to “very important/very reasonable/very feasible,” with values assigned on a scale from 1 to 5. In addition, each indicator was accompanied by a column for expert’s modification suggestions. The third part was the expert’s basic information (age, education level, professional title), familiarity with the indicators, and judgment basis.

### Selection of experts

In accordance with the principles of representativeness and authority, the number of experts chose for the Delphi study ranged from 15 to 50 [24]. The inclusion criteria for experts were (a): bachelor degree or above; (b):professional title of intermediate or above; (c): ≥5 years of work experience; (d): engaged in clinical obstetric nursing, nursing quality management, and evidence-based nursing in obstetrical department at tertiary hospitals; (e) willingly participate and complete the questionnaire timely.

### Questionnaire distribution

Eighteen experts from national tertiary hospitals in seven provinces and cities in China participated in the consultations. A two-tier Delphi study was conducted between July and August 2024. After obtaining informed consent from the experts, the research group distributed questionnaires via WeChat or email and requested that they complete and return each round of questionnaires within two weeks. Following the initial round of inquiries, two face-to-face group discussions were conducted by members of the research team.All indicators were modified or deleted based on the criteria of mean value ≥ 4.00, full score ratio ≥ 20% and coefficient of variation (CV) ≤0.25 [25]. Furthermore, expert opinions and the results of group discussions were utilized to either remove or modify indicators in order to generate the subsequent round of inquiry questionnaires. A total of two rounds of inquiries were conducted in this study.

### Analytic hierarchy process

Analytic hierarchy process (AHP) was used to confirm the weight of each indicator [26]. Based on the results of the second round of Delphi study, a hierarchical structure consisting of target layer, criterion layer, and scheme layer was constructed. The target layer in this study was NSQIs system for PA-VTE, the criterion layer in this study referred to the levels determined to achieve predetermined goals, including primary and secondary indicators. The scheme layer in this study was solutions developed to achieve predetermined goals, including tertiary indicator. Besides, we compared the indicators at each level in pairs based on the hierarchical structure. The Saaty 1–9 scale was determined by the mean difference in the importance ratings assigned by the expert panel in the second round of consultation.

### Statistical analysis

All the data were managed and analysed by the social science statistical software package SPSS (Mac version 20.0). Quantitative data that accorded with normal distribution were expressed as mean and standard deviation (x±s), while qualitative data were expressed as frequency and percentage (%). The positive coefficient of Delphi experts was expressed by calculating the recovery rate of the consultation form. The authority of experts is represented by the authority coefficient (*Cr*). The degree of coordination of expert opinions is represented by coefficient of variation (*CV*) and coordination coefficient (Kendall *W*). Indicators’ weight values and consistency test values were calculated via Mesh AHP software V1.82.

### Ethics considerations

The study was approved by the Foshan Women and Children Hospital Ethics Committee (Application number:FSFY-MEC-2023–043). Information about the study was provided to participants in the Participant Information Sheet. Eligible participants were required to sign a consent form, and were informed of their right to withdraw from the study at any time. Furthermore, the identities and identifiable information of the participants will be kept strictly confidential.

## Results

### Characteristics of expert

Eighteen experts from 11 tertiary hospitals located in 7 provinces and cities in China participated in the consultations. Most experts were senior nurses with more than 10 years of clinical experience, which supports the authority and representativeness of the panel. Table 1 describes the expert’s general characteristics.

**Table 1:** Experts’ general characteristics (*n* = 18)

Variables	N(f)	Percentage (%)
Age (years)		
≤40	5	28
41–50	10	56
≥51	3	16
Gender		
Male	3	16
Female	15	84
Degree of education		
Undergraduate	12	67
Master’s	4	22
Doctorate	2	11
Title		
Intermediate	5	28
Deputy senior staff	9	50
Senior staff	4	22
Years of working		
≤10	3	16
11–20	7	39
≥21	8	45

### Reliability of the Delphi method

#### Experts’ positive coefficients and authority coefficient

The recovery rate was 100% for both study rounds. The *Ca*, *Cs* and *Cr* values determined in this study were 0.98, 0.81, and 0.89, respectively, A Cr value of 0.89 indicates a high level of expert authority and supports the credibility of the Delphi results.

#### Coordination level of experts’ opinions

In the first round of expert consultation, *W* values indicating that the indicator’s importance, formula rationality, and method applicability were 0.115, 0.093 and 0.114, respectively. In the second round, they were 0.136,0.096 and 0.100, respectively. A *p*-value < 0.05 indicated that the experts reached a consensus. Although Kendall’s W values indicate a relatively low-to-moderate level of agreement, all were statistically significant. These indices are interpreted together with the mean scores, coefficients of variation, and full-score ratios, suggesting an acceptable level of consensus among experts. Details are shown in Table 2.

**Table 2:** Coordination level of experts’ opinions

Project	Round 1			Round 2		
	Kendall’s(ω)	**X**^**2**^ **value**	P-value	Kendall’s(ω)	**X**^**2**^**value**	P-value
Indicator’s importance	0.115	78.777	＜0.001	0.136	93.336	＜0.001
Indicator formula’s rationality	0.093	48.336	0.014	0.096	43.243	0.013
Method’s applicability	0.114	54.495	0.001	0.100	44.919	0.009

#### Indicator modifications

In the first round, one secondary and five tertiary indicators were deleted, 13 tertiary indicators were merged into four broader indicators, and three new secondary indicators and 15 new tertiary indicators were added. After the group discussion, the secondary indicator “nursing efficacy” and 5 tertiary indicators were removed. Additionally, 13 tertiary indicators were combined into 4 new ones, such as merging “rate of qualified liquid intake”, “rate of compliance of ankle pump movement”, and “rate of compliance of early mobilization” into new tertiary indicator named “rate of administration of basic preventive measures”. Three secondary indicators were modified, such as changing the “equipment configuration” to “resources and equipment” and “nursing assessment” to “risk assessment”. Furthermore, three new secondary indicators - namely, “health education”, “nursing records” and “quality of service”- along with 15 tertiary indicators like “rate of implementation of nursing management system for VTE prevention”, “rate of accurate nursing records on VTE prevention” were added (see Table 3 for details).

**Table 3:** The proposed indicators and their revisions after the two rounds of expert consultation

Indicator	Round 1					Round 2				
	M±SD	CV	Percentage for full score(%)	Outcome	Revised or added indicator	M±SD	CV	Percentage for full score(%)	Outcome	Revised indicator
1.Structure	4.78 ± 0.42	0.09	77.8	Accepted		4.89 ± 0.31	0.06	88.9	Accepted	
1.1 Management system	4.83 ± 0.37	0.08	77.8	Accepted		4.89 ± 0.31	0.06	88.9	Accepted	
1.1.1 Rate of implementation of regular training for VTE prevention and control team	4.67 ± 0.47	0.10	66.7	Deleted	Rate of implementation of nursing management system for VTE prevention	4.83 ± 0.37	0.08	83.3	Accepted	
1.1.2 Rate of qualified management of VTE equipment	4.56 ± 0.50	0.11	77.8	Deleted	Rate of implementation of nursing standardization procedure for VTE prevention	4.83 ± 0.37	0.08	83.3	Accepted	
					Rate of implementation of assessment system for VTE risk	4.83 ± 0.37	0.08	83.3	Accepted	
					Ratio of obstetric specialist nurses for VTE prevention	4.61 ± 0.49	0.11	61.1	Accepted	
1.2 Equipment configuration	4.39 ± 0.68	0.15	50	Revised	Resources and equipment	4.78 ± 0.53	0.11	83.3	Accepted	
1.2.1 Rate of usage of gradient pressure elastic socks	4.33 ± 0.88	0.20	55.6	Revised	Rate of completeness of equipment for VTE prevention	4.61 ± 0.59	0.13	66.7	Accept	
1.2.2 Rate of usage of intermittent pneumatic compression	4.72 ± 0.45	0.09	72.2							
1.2.3 Rate of usage of sole vein pump	4.44 ± 0.68	0.15	55.6							
					Rate of completeness of assessment tool for VTE prevention	4.44 ± 0.60	0.13	61.1	Deleted	
					Rate of completeness of health education materials for VTE prevention	4.44 ± 0.60	0.13	50	Accepted	
1.3 Teaching and training	4.67 ± 0.47	0.10	66.7	Accepted		4.89 ± 0.31	0.06	88.9	Accepted	
1.3.1 Rate of qualified test of VTE theoretical knowledge among obstetric nurses	4.56 ± 0.50	0.11	55.6	Accepted		4.56 ± 0.50	0.13	55.6	Accepted	
1.3.2 Rate of qualified test of operational skills for VTE prevention among obstetric nurses	4.50 ± 0.50	0.11	50	Accepted		4.56 ± 0.50	0.11	55.6	Accepted	
					Rate of implementation of regular training for VTE team	4.56 ± 0.50	0.11	55.6	Accepted	
2.Process	4.89 ± 0.31	0.06	88.9	Accepted		4.89 ± 0.31	0.06	88.9	Accepted	
2.1 Nursing assessment	4.72 ± 0.45	0.09	72.2	Revised	Risk assessment	4.83 ± 0.50	0.10	88.9	Accepted	
2.1.1 Rate of assessment of VTE risk within 24 hours after admission	4.50 ± 0.60	0.13	55.6	Deleted	Rate of timely assessment of VTE risk for pregnant and postpartum women	4.67 ± 0.58	0.12	72.2	Accepted	
2.1.2 Rate of assessment of VTE risk within 24 hours before surgery	4.39 ± 0.76	0.17	55.6							
2.1.3 Rate of assessment of VTE risk within 24 hours after surgery	4.56 ± 0.60	0.13	61.1							
2.1.4 Rate of assessment of VTE risk within 24 hours after transfer	4.44 ± 0.60	0.13	50							
2.1.5 Rate of assessment of VTE risk within 24 hours before discharge	4.39 ± 0.68	0.15	50							
					Rate of accurate assessment content of VTE risk for pregnant and postpartum women	4.72 ± 0.45	0.09	72.2	Revised	Rate of assessment of VTE risk for pregnant and postpartum women
2.1.6 Rate of implementation of the first assessment of VTE bleeding risk for pregnant and postpartum women	4.56 ± 0.50	0.11	55.6	Accepted		4.61 ± 0.49	0.11	61.1	Accepted	
2.1.7 Rate of implementation of re-assessment of VTE bleeding risk for pregnant and postpartum women	4.50 ± 0.60	0.13	55.6	Accepted		4.56 ± 0.50	0.11	55.5	Accepted	
					Health education	4.78 ± 0.53	0.11	83.3	Accepted	
					Rate of education on VTE prevention for pregnant and postpartum women	4.61 ± 0.59	0.13	66.7	Accepted	
					Rate of accurate education contents on VTE prevention for pregnant and postpartum women	4.56 ± 0.83	0.18	72.2	Accepted	
					Rate of timely education on VTE prevention for pregnant and postpartum women	4.33 ± 1.00	0.23	61.1	Accepted	
2.2 Nursing measures	4.78 ± 0.42	0.09	77.8	Revised	Preventive measures	4.78 ± 0.53	0.11	83.3	Accepted	
2.2.1 Rate of qualified liquid intake	4.67 ± 0.47	0.10	66.7	Deleted	Rate of administration of basic preventive measures	4.72 ± 0.45	0.09	72.2	Accepted	
2.2.2 Rate of compliance of ankle pump movement	4.72 ± 0.45	0.09	72.2							
2.2.3 Rate of compliance of early mobilization	4.67 ± 0.58	0.12	72.2							
2.2.4 Rate of accurate usage of gradient pressure elastic socks	4.39 ± 0.59	0.13	44.4		Rate of administration of mechanical preventive measures	4.67 ± 0.47	0.10	66.7	Accepted	
2.2.5 Rate of accurate usage of intermittent pneumatic compression	4.44 ± 0.60	0.13	50							
2.2.6 Rate of usage of sole vein pump	4.56 ± 0.60	0.13	61.1							
2.2.7Rate of accurate administration of anticoagulant drugs	4.56 ± 0.60	0.13	61.1		Rate of administration of drug prophylaxis measures	4.61 ± 0.49	0.11	61.1	Accepted	
2.2.8Rate of monitoring of adverse reaction for anticoagulant drugs	4.72 ± 0.45	0.09	72.2							
2.2.9 Rate of education on VTE prevention within 24 hours after admission	4.67 ± 0.47	0.10	66.7	Deleted						
2.2.10 Rate of education on VTE prevention after surgery	4.67 ± 0.47	0.10	66.7	Deleted						
2.2.11 Rate of education on VTE prevention before discharge	4.61 ± 0.49	0.11	61.1	Deleted						
					Nursing records	4.56 ± 0.83	0.18	72.2	Accepted	
					Rate of accurate nursing documentation on VTE prevention	4.56 ± 0.60	0.13	61.1	Accepted	
3.Outcome	4.83 ± 0.37	0.08	83.3	Accepted		4.89 ± 0.31	0.06	0.89	Accepted	
3.1Outcomes of pregnant and postpartum women	4.78 ± 0.42	0.09	77.8	Accepted		4.69 ± 0.49	0.11	61.1	Accepted	
3.1.1 Incidence of hospital-acquired VTE	4.61 ± 0.49	0.11	61.1	Accepted		4.67 ± 0.47	0.10	66.7	Accepted	
3.1.2 Incidence of bleeding	4.61 ± 0.49	0.11	61.1	Deleted	Incidence of complications related to VTE prevention measures	4.56 ± 0.50	0.11	61.1	Accepted	
					Incidence of adverse reactions related to anticoagulation	4.56 ± 0.50	0.11	61.1	Accepted	
3.2 Nursing efficacy	4.72 ± 0.45	0.09	72.2	Revised	Cognitive behavior of pregnant and postpartum women	4.50 ± 0.60	0.13	55.6	Accepted	
3.2.1 Rate of knowledge of VTE prevention on pregnant and postpartum women	4.67 ± 0.47	0.10	66.7	Accepted		4.61 ± 0.49	0.11	61.1	Accepted	
					Quality of service	4.44 ± 0.68	0.15	55.6	Deleted	
3.2.2 Satisfaction of pregnant and postpartum women	4.28 ± 0.65	0.15	61.1	Accepted		4.39 ± 0.68	0.15	50	Deleted	

In the second round, one secondary and two tertiary indicators were deleted, one tertiary indicator was revised. Following the incorporation of expert opinions, the group excluded the secondary indicator “quality of service” and the tertiary indicators “satisfaction of pregnant and postpartum women” and “rate of completeness of assessment tool for VTE prevention” from the evaluation index. The tertiary indicator “rate of accurate assessment content of VTE risk for pregnant and postpartum women” was revised to “rate of assessment of VTE risk for pregnant and postpartum women.” Additionally, adjustments were made to the calculation formulas for certain indicators.

The results of the two survey rounds are presented in Tables 3 and 4. The final NSQIs are consisted of 3 primary indicators, 9 secondary indicators, and 24 tertiary indicators.

**Table 4:**
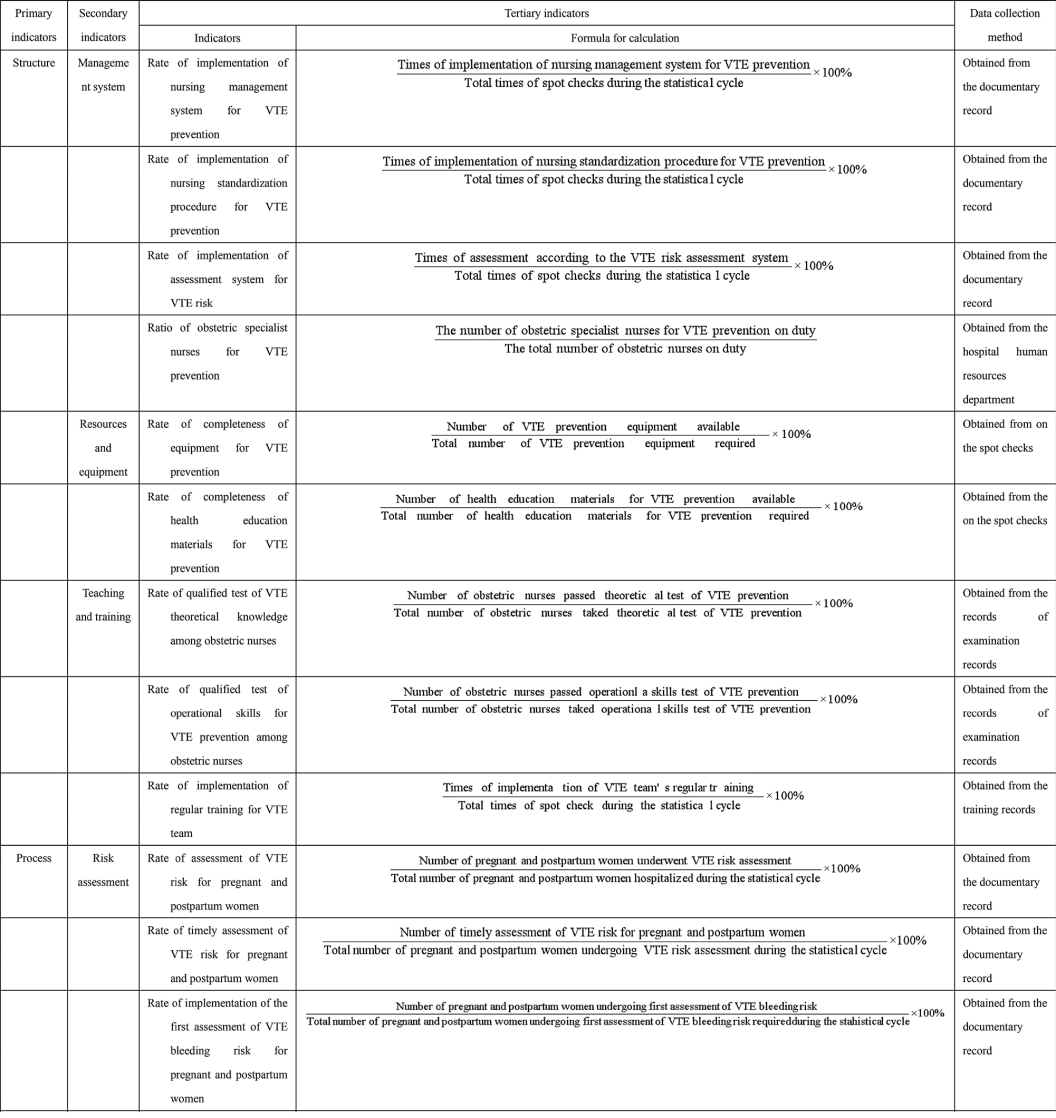

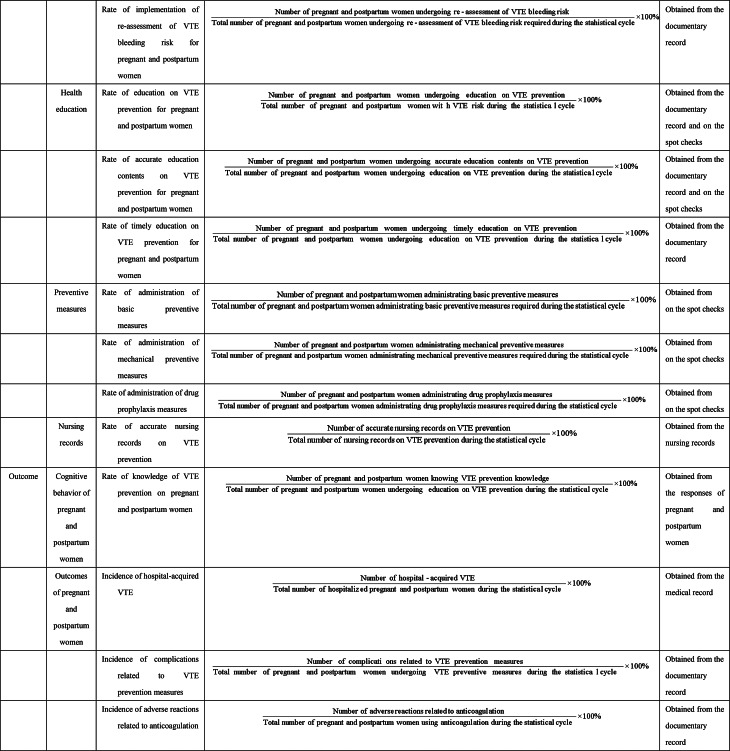
Nursing-sensitive quality indicators for pregnancy-associated venous thromboembolism

#### Relative importance of the indicators

The relative importance weight of each indicator is shown in Table 5. At the primary level, structure and process dimensions had equal weights (0.40 each), both higher than the outcome dimension (0.20). Among the secondary indicators, the outcomes of pregnant and postpartum women (0.2500), risk assessment (0.1800) and management system (0.1526) were the top three indicators. Among the tertiary indicators, the rate of assessment of VTE risk for pregnant and postpartum women (0.1517), incidence of hospital-acquired VTE (0.0667) and rate of implementation of nursing management system for VTE prevention (0.0603) were the top three indicators.These results indicate that structural capacity and process quality, particularly risk assessment and management systems, are viewed by experts as slightly more critical than outcome indicators in evaluating PA-VTE nursing quality.

**Table 5:** Relative importance of quality indicators for pregnancy-associated venous thromboembolism (rank by weight)

	Indicator	Weight
Primary indicators	Process	0.4000
	Structure	0.4000
	Outcome	0.2000
Secondary indicators	Outcomes of pregnant and postpartum women	0.2500
	Risk assessment	0.1800
	Management system	0.1526
	Teaching and training	0.1387
	Cognitive behavior of pregnant and postpartum women	0.0833
	Preventive measures	0.0637
	Health education	0.0637
	Resources and equipment	0.0420
	Nursing records	0.0193
Tertiary indicators	Rate of assessment of VTE risk	0.1517
	Incidence of hospital-acquired VTE	0.0667
	Rate of implementation of nursing management system for VTE prevention	0.0603
	Rate of implementation of nursing standardization procedure for VTE prevention	0.0603
	Rate of implementation of assessment system for VTE risk	0.0603
	Rate of implementation of the first assessment of VTE bleeding risk	0.0587
	Rate of accurate nursing records on VTE prevention	0.05
	Rate of implementation of re-assessment of VTE bleeding risk	0.0493
	Rate of qualified test of operational skills for VTE prevention	0.0443
	Rate of implementation of regular training for VTE team	0.0443
	Rate of qualified test of VTE theoretical knowledge	0.0443
	Rate of completeness of VTE prevention equipment	0.0419
	Rate of administration of basic preventive measures	0.0351
	Incidence of complications related to VTE prevention measures	0.0333
	Incidence of adverse reactions related to anticoagulation	0.0333
	Rate of education on VTE prevention	0.0319
	Ratio of obstetric specialist nurses for VTE prevention	0.0302
	Rate of timely assessment of VTE risk	0.0261
	Rate of accurate education contents on VTE prevention	0.0183
	Rate of knowledge of VTE prevention	0.0167
	Rate of administration of mechanical preventive measures	0.0153
	Rate of completeness of health education materials for VTE prevention	0.014
	Rate of timely education on VTE prevention	0.007
	Rate of administration of drug prophylaxis measures	0.0067

## Discussion

Based on the Donabedian model and through a rigorous, evidence-based Delphi process, this study finally developed a set of NSQIs for PA-VTE, which included 3 primary indicators, 9 secondary indicators and 24 tertiary indicators. Among these, structure and process dimensions had the highest weights, and risk assessment and maternal outcomes emerged as the most influential domains.

The structural indicators established in this study are crucial for maintaining the quality of nursing care in preventing PA-VTE, including three secondary indicators named management system (0.1526), resources and equipment (0.0420), and teaching and training (0.1387), as well as nine tertiary indicators.The management system had the third-highest weight among secondary indicators (0.1526), following outcomes of pregnant and postpartum women and risk assessment. Among tertiary indicators, rate of implementation of nursing management system for VTE prevention, rate of implementation of nursing standardization procedure for VTE prevention and rate of implementation of assessment system for VTE risk have equal weights (0.0603). This highlights its crucial role in PA-VTE prevention. Improving the management of standardized systems and optimizing processes is essential for preventing PA-VTE, and it plays a crucial role in decreasing the occurrence of PA-VTE [27]. Although the weight of the tertiary indicator, ratio of obstetric specialist nurses for VTE prevention, is relatively low, the core competency level of specialized nurses to a certain extent represents the quality of specialized nursing services. Our results underscore the need to establish an evaluation system for the core competencies of specialized nurses in obstetric VTE prevention. Teaching and training are essential components of quality improvement measures. The proficiency of nurses in technical skills and theoretical knowledge directly influences the execution quality of PA-VTE prevention measures. A multicenter survey conducted among nurses in China revealed suboptimal mastery of knowledge related to VTE prevention [28, 29], with an observed imbalance in knowledge acquisition, particularly among obstetric and gynecological nurses [28]. Studies have shown that standardized regular training can improve nurses’ theoretical knowledge of VTE prevention [30]. This suggests the need for nursing managers to establish obstetric VTE prevention and control teams, as well as develop a nurse training system guided by job demands and core competencies. Additionally, resources and equipment, including VTE prevention devices and health education materials, are essential guarantees for implementing preventive measures. The mechanical and pharmacological adherence to VTE prophylaxis among hospitalized patients is low [31], which may be related to the insufficiency of VTE prevention devices and health education materials [32, 33]. Therefore, it is necessary to improve the in-hospital product configuration rate and enhance the diversity of educational materials.

Process indicators ensure the accurate execution of nursing practices and are a key component of the quality of PA-VTE preventive nursing care, including four secondary indicators of risk assessment (0.1800), health education (0.0637), preventive measures (0.0637), and nursing records (0.0193), and 11 tertiary indicators. The risk assessment indicator has the highest weight (0.1800), while among the tertiary indicators, the VTE risk assessment rate ranks first with a weight of 0.1517. In accordance with guidelines [34], the scientific and timely nature of VTE risk assessment for pregnant women significantly influences nurses’ clinical judgment. VTE exhibits a high incidence and a significant rate of underdiagnosis, particularly within the specific demographic of pregnant women. Therefore, the utilization of appropriate VTE risk assessment tools [35] to precisely identify early VTE and bleeding risks, as well as conducting risk stratification, is essential for implementing targeted preventive measures. A nationwide survey conducted in Germany [36] has indicated a low rate of VTE risk assessment among pregnant women. This underscores the need for nursing manager to intensify training for nurses on dynamic VTE risk assessment in pregnant women and the proper utilization of existing assessment tools.

Furthermore, health beliefs are a prerequisite for promoting healthy behaviors [37], and the occurrence and development of VTE are closely associated with the lifestyle and adherence to medical advice among pregnant women. Studies have shown that pregnant women have poor compliance with VTE prevention measures [38, 39], with 89% of them reporting a lack of understanding about VTE and expressing the need for health education from healthcare providers [40]. Nursing staff in clinical practice need to prioritize providing pregnant women with health education on VTE prevention, including the timing and accuracy of the content. This can promote the adoption of healthy lifestyles among pregnant women, enhance self-awareness and management skills, and lay a foundation for the successful implementation of VTE prevention measures.

Implementing risk-stratified preventive measures is an effective safeguard for reducing the incidence of PA-VTE [41]. The implementation of a comprehensive VTE prevention strategy can significantly reduce maternal mortality rates [42]. However, the selection of preventive measures should take into account factors such as cost, benefits, and risk assessment levels for pregnant women [43, 44]. To ensure applicability across hospitals with varying resources, experts emphasized that developed indicators should be universal. Therefore, rather than fine-tuning the types of preventive measures as quality indicators, only the rate of administration of basic preventive measures, rate of administration of mechanical preventive measures and rate of administration of drug prophylaxis measures were used as the tertiary indicators to monitor the quality of VTE preventive measures. Furthermore, the secondary indicator of nursing record should not be overlooked. The standardized nursing records of PA-VTE prevention is essential as it serves as a crucial reference for nursing practice and holds significant legal importance.

The outcome indicators are specific and focus on the quality of VTE preventive nursing care, including two secondary indicators (outcomes of pregnant and postpartum women, cognitive behavior of pregnant and postpartum women) and four tertiary indicators. Among all tertiary indicators, incidence of hospital-acquired VTE (0.0667) and incidence of complications related to VTE prevention measures (0.0333) were the highest-weighted outcome indicators.

The process indicators exert a direct influence on the outcome indicators. Nurses, as the primary agents of nursing practice, play a crucial role in facilitating accurate implementation of risk assessment and preventive measures. The tertiary indicators, including the incidence of hospital-acquired VTE, incidence of complications related to VTE prevention measures and incidence of adverse reactions related to anticoagulation, are important indicators for evaluating obstetric nurses’ capability in VTE prevention. Additionally, the rate of knowledge of VTE prevention on pregnant and postpartum women serves as feedback on nursing services and health education practices, effectively evaluating the quality of nursing care.

To the best of our knowledge, this study is the first to develop a set of NSQIs specifically targeted at women with PA-VTE. Zhu et al. [45] constructed 16 obstetric nursing-sensitive quality indicators through the improved Delphi method, among which only one - the number of cases of deep vein thrombosis in pregnant and postpartum women- is related to VTE. This not only reflects the insufficient attention paid to PA-VTE in the existing obstetric indicator system, but also exposes the flaw that relevant indicators only focus on outcomes while lacking the support of process-oriented dimensions. In response to this situation, the NSQIs for PA-VTE constructed in this study can effectively make up for the deficiencies of the existing system, and provide a more systematic and operable professional tool for hospital managers to carry out benchmarking, auditing and quality improvement of PA-VTE.

## Limitations

The NSQIs developed in this study have several limitations that should be acknowledged. First, aggregated indicators such as “management system” and “resources and equipment” have clear calculation formulas, but their operational definitions still lack fully objective quantitative standards, which may introduce variability in local implementation. Second, all Delphi experts were recruited from tertiary hospitals in China. This may limit the generalizability of the indicator system to lower-level hospitals or to other countries. Third, both the Delphi ratings and the AHP-derived weights rely on expert opinion rather than empirical outcome data, and crucially, the NSQIs have not yet undergone testing for feasibility, reliability, and sensitivity in the context of routine clinical data. Finally, although we provided detailed guidance on how to apply each indicator (Supplementary Table 1), the broad scope of some aggregated indicators leaves room for differences in interpretation among data collectors.

## Conclusion

Using Donabedian’s structure–process–outcome framework, this study developed a set of 3 primary, 9 secondary, and 24 tertiary nursing-sensitive quality indicators specifically for pregnancy-associated venous thromboembolism. These indicators are quantifiable and clinically meaningful, providing a structured tool for monitoring nursing quality, guiding targeted quality improvement initiatives, and promoting standardization within specialized obstetric nursing. Future work should focus on implementing these indicators in different hospital settings, evaluating their feasibility and reliability in routine data collection, and refining the indicator set based on empirical performance and patient outcomes.

## Electronic supplementary material

Below is the link to the electronic supplementary material.

Supplementary Material 1

Supplementary Material 2

## Data Availability

All data generated or analysed during this study are included in this published article.
