# Supplementary material for: Construction of nursing-sensitive quality indicators for pregnancy-associated venous thromboembolism using the Delphi method
Source: BMC Nurs. 2026 Mar 7;25:358. doi: 10.1186/s12912-026-04517-y (PMC13081401; doi:10.1186/s12912-026-04517-y)
Supplement: Supplementary file 1 — Supplementary Material 1 [file 12912_2026_4517_MOESM1_ESM.docx]

**Supplementary Appendix 1**

**A Delphi questionnaire on**

**construction of nursing-sensitive quality indicators for pregnancy-associated venous thromboembolism**

Dear experts:

Thank you very much for taking time to participate in our study!

Our team is currently conducting a study on the construction of nursing-sensitive quality indicators for pregnancy-associated venous thromboembolism. Pregnancy-associated venous thromboembolism (PA-VTE) refers to a specific type of venous thromboembolism (VTE) that arises from the interplay of obstetric and non-obstetric factors, influenced by the unique physiological conditions present during pregnancy and the puerperium. The risk of VTE among pregnant and postpartum women is 3-4 times higher than that of non-pregnant women, seriously affecting their life health and quality of life. The quality level of nursing services affects the disease progression of VTE pregnant and postpartum women. According to the literature review, however, there is no nursing-sensitive quality indicator system for PA-VTE in China. Given that, the study aims to establish a scientific nursing sensitive quality indicator system for PA-VTE based on evidence practice, in order to promote continuous improvement of nursing quality in clinical practice.

Our team now sincerely invite you to review the Delphi questionnaire, which contains two parts. Please send the questionnaire to us within 1 week. If you have any questions, feel free to contact us at any time. We will strictly keep your relevant information confidential. Thank you again for your cooperation

**Part one Draft of constructed nursing-sensitive quality indicators for pregnancy-associated venous thromboembolism**

The preliminary proposed draft covers primary, secondary and tertiary indicators:

1. Please judge the importance degree of indicators, rationality of calculation formula, feasibility of operational methods and mark "√" in the columns.

（2）If you have any modifications to the indicators, please fill them out in the "Revision recommendations" column.

（3）Please add or delete the entries you think are necessary in the "Added / deleted entries".

**A Delphi questionnaire on primary indicators**

| Primary indicators | Importance | | | | | Revision recommendations |
| --- | --- | --- | --- | --- | --- | --- |
|  | Very important  (5 points) | Important  (4 points) | General important  (3 points) | Not very important  (2 points) | Very unimportant  (1 point) |  |
| I-1 Structure |  |  |  |  |  |  |
| I-2 Process |  |  |  |  |  |  |
| I-3 Outcome |  |  |  |  |  |  |

**A Delphi questionnaire on secondary indicators**

| Primary indicators | Secondary indicators | Is it associated with  the appropriate category?  (Yes/No) | Importance | | | | | Revision recommendations |
| --- | --- | --- | --- | --- | --- | --- | --- | --- |
|  |  |  | Very  important  (5 points) | Important  (4 points) | General important  (3 points) | Not very important  (2 points) | Very unimportant  (1 point) |  |
| I-1 Structure | II-1 Management system |  |  |  |  |  |  |  |
|  | II-2 Equipment configuration |  |  |  |  |  |  |  |
|  | II-3 Teaching and training |  |  |  |  |  |  |  |
|  | Added / Deleted entries |  |  |  |  |  |  |  |
| I-2 Process | II-4 Nursing assessment |  |  |  |  |  |  |  |
|  | II-5 Nursing measures |  |  |  |  |  |  |  |
|  | Added / Deleted entries |  |  |  |  |  |  |  |
| I-3 Outcome | II-6 Outcomes of pregnant and postpartum women |  |  |  |  |  |  |  |
|  | II-7 Nursing efficacy |  |  |  |  |  |  |  |
|  | Added / Deleted entries |  |  |  |  |  |  |  |

**A Delphi questionnaire on tertiary indicators**

| Secondary indicators | Tertiary indicators | Is it associated with  the appropriate category?  (Yes/No) | Importance | | | | | Revision recommendations | Calculation formula | Reasonbility | | | | | Revision recommendations | Feasibility | | | | | Revision recommendations |
| --- | --- | --- | --- | --- | --- | --- | --- | --- | --- | --- | --- | --- | --- | --- | --- | --- | --- | --- | --- | --- | --- |
|  |  |  | a | b | c | d | e |  |  | f | g | h | i | j |  | k | l | m | n | o |  |
| II-1  Management system | III-1  Implementation rate of regular training for VTE prevention and control team |  |  |  |  |  |  |  | **=** Times of team training for VTE prevention / Total times of team training for VTE prevention required x100% |  |  |  |  |  |  |  |  |  |  |  |  |
|  | III-2  Qualified rate of VTE equipment management |  |  |  |  |  |  |  | = Times of qualified inspections for VTE equipment management / Total times of inspections conducted for VTE equipment management during the statistical cycle x100% |  |  |  |  |  |  |  |  |  |  |  |  |
| II-2  Equipment configuration | III-3  Usage rate of gradient pressure stockings |  |  |  |  |  |  |  | = Number of cases using gradient pressure stockings / Total number of cases using gradient pressure stockings during the statistical cycle x100% |  |  |  |  |  |  |  |  |  |  |  |  |
|  | III-4  Usage rate of intermittent inflation pressure device |  |  |  |  |  |  |  | = Number of cases using intermittent inflation pressure device / Total number of cases using intermittent inflation pressure device during the statistical cycle x100% |  |  |  |  |  |  |  |  |  |  |  |  |
|  | III-5  Usage rate of foot vein pump |  |  |  |  |  |  |  | = Number of cases using foot vein pump / Total number of cases using foot vein pump during the statistical cycle x100% |  |  |  |  |  |  |  |  |  |  |  |  |
| II-3  Teaching and training | III-6  Rate of qualified test of theoretical knowledge for VTE prevention among obstetric nurses |  |  |  |  |  |  |  | = Number of obstetric nurses passed theoretical test of VTE prevention / Total number of obstetric nurses took theoretical test of VTE prevention x100% |  |  |  |  |  |  |  |  |  |  |  |  |
|  | III-7  Rate of qualified test of operational skills for VTE prevention among obstetric nurses |  |  |  |  |  |  |  | = Number of obstetric nurses passed operational test of VTE prevention / Total number of obstetric nurses took operational test of VTE prevention x100% |  |  |  |  |  |  |  |  |  |  |  |  |
| II-4  Nursing assessment | III-8  Rate of assessment of VTE risk within 24 hours of admission |  |  |  |  |  |  |  | = Number of pregnant and postpartum women undergoing VTE risk assessment within 24 hours of admission / Total number of pregnant and postpartum women undergoing VTE risk assessment within 24 hours of admission required during the statistical cycle x100% |  |  |  |  |  |  |  |  |  |  |  |  |
|  | III-9  Rate of assessment of VTE risk within 24 hours prior to surgery |  |  |  |  |  |  |  | = Number of pregnant women undergoing VTE risk assessment within 24 hours prior to surgery / Total number of pregnant women undergoing VTE risk assessment within 24 hours prior to surgery required during the statistical cycle x100% |  |  |  |  |  |  |  |  |  |  |  |  |
|  | III-10  Rate of assessment of VTE risk within 24 hours after surgery |  |  |  |  |  |  |  | = Number of postpartum women undergoing VTE risk assessment within 24 hours after surgery / Total number of women who had cesarean deliveries during the statistical cycle x100% |  |  |  |  |  |  |  |  |  |  |  |  |
|  | III-11  Rate of assessment of VTE risk within 24 hours post-transfer to another department |  |  |  |  |  |  |  | = Number of pregnant and  postpartum women undergoi-  ng VTE risk assessment  within 24 hours post-transfer  to another department / Total  number of pregnant and  postpartum women undergoi-  ng VTE risk assessment  within 24 hours post-transfer  to another department  required during the statistical  cycle x100% |  |  |  |  |  |  |  |  |  |  |  |  |
|  | III-12  Rate of assessment of VTE risk within 24 hours prior to discharge |  |  |  |  |  |  |  | = Number of pregnant and postpartum women undergoing VTE risk assessment within 24 hours prior to discharge / Total number of pregnant and postpartum women undergoing VTE risk assessment within 24 hours prior to discharge required during the statistical cycle x100% |  |  |  |  |  |  |  |  |  |  |  |  |
|  | III-13  Rate of implementation of the first assessment of VTE bleeding risk for pregnant and postpartum women |  |  |  |  |  |  |  | = Number of pregnant and  postpartum women  undergoing first  assessment of VTE bleeding  risk / Total number of  pregnant and postpartum  women with high or moderate  risk of VTE during the  statistical period x100% |  |  |  |  |  |  |  |  |  |  |  |  |
|  | III-14  Rate of implementation of re-assessment of VTE bleeding risk for pregnant and postpartum women |  |  |  |  |  |  |  | = Number of pregnant and  postpartum women  undergoing re-assessment of  VTE bleeding risk / Total  number of pregnant and  postpartum women with high  or moderate risk of VTE  during the statistical period  x100% |  |  |  |  |  |  |  |  |  |  |  |  |
| II-5  Nursing measures | III-15  Rate of correct usage of gradient pressure stockings |  |  |  |  |  |  |  | = Number of cases correctly using gradient pressure stockings / Total number of cases using gradient pressure stockings during the statistical cycle x100% |  |  |  |  |  |  |  |  |  |  |  |  |
|  | III-16  Rate of correct usage of intermittent inflation pressure device |  |  |  |  |  |  |  | = Number of cases correctly using intermittent inflation pressure device / Total number of cases using intermittent inflation pressure device during the statistical cycle x100% |  |  |  |  |  |  |  |  |  |  |  |  |
|  | III-17  Rate of correct usage of foot vein pump |  |  |  |  |  |  |  | = Number of cases correctly using foot vein pump / Total number of cases using foot vein pump during the statistical cycle x100% |  |  |  |  |  |  |  |  |  |  |  |  |
|  | III-18  Rate of correct  administration of anticoagulant drugs |  |  |  |  |  |  |  | = Times of correctly  administration of anticoagul-  ant drugs / Times of administration of anticoagul-  ant drugs during the statistical cycle x100% |  |  |  |  |  |  |  |  |  |  |  |  |
|  | III-19  Execution rate of monitoring adverse reactions of anticoagulant drugs |  |  |  |  |  |  |  | = Number of cases monitored for adverse effects of anticoagulant drugs / Total numbers of cases took anticoagulant drugs for prevention during the statistical cycle x100% |  |  |  |  |  |  |  |  |  |  |  |  |
|  | III-20  Qualified rate of fluid management |  |  |  |  |  |  |  | = Number of cases with adequate daily fluid intake / Total number of cases with VTE risk during the statistical period x100% |  |  |  |  |  |  |  |  |  |  |  |  |
|  | III-21  Rate of compliance of ankle pump exercise |  |  |  |  |  |  |  | **=** Number of cases who actually performed ankle pump exercises / Total number of cases who performed ankle pump exercises required during the statistical period x100% |  |  |  |  |  |  |  |  |  |  |  |  |
|  | III-22  Rate of compliance of early activity |  |  |  |  |  |  |  | **=** Number of cases who actually performed early activity/ Total number of cases who performed early activity required during the statistical period x100% |  |  |  |  |  |  |  |  |  |  |  |  |
|  | III-23  Rate of implementation of education on VTE prevention  within 24 hours of admission |  |  |  |  |  |  |  | = Number of cases who received education on VTE prevention within 24 hours of admission / Total number of cases with VTE risk during the statistical period x100% |  |  |  |  |  |  |  |  |  |  |  |  |
|  | III-24  Rate of implementation of education on VTE prevention  within 24 hours after surgery |  |  |  |  |  |  |  | = Number of cases who received education on VTE prevention within 24 hours after surgery / Total number of women who had cesarean deliveries during the statistical cycle x100% |  |  |  |  |  |  |  |  |  |  |  |  |
|  | III-25  Rate of implementation of education on VTE prevention  within 24 hours before discharge |  |  |  |  |  |  |  | = Number of cases who received education on VTE prevention within 24 hours before discharge / Total number of cases with VTE risk during the statistical period x100% |  |  |  |  |  |  |  |  |  |  |  |  |
| II-6  Outcome | III-26  Incidence of in-hospital VTE |  |  |  |  |  |  |  | = Number of cases who diagnosed with hospital-  acquired VTE / Total number of cases who discharged within 90 days after staying in the hospital for more than 2 days during the statistical period x100% |  |  |  |  |  |  |  |  |  |  |  |  |
|  | III-27  Incidence of bleeding |  |  |  |  |  |  |  | **=** Number of cases with bleeding / Total number of cases received anticoagulant drugs during the statistical period x100% |  |  |  |  |  |  |  |  |  |  |  |  |
| II-7  Nursing efficacy | III-28  Rate of knowledge of VTE prevention on pregnant and postpartum women |  |  |  |  |  |  |  | = Number of cases knowing VTE prevention knowledge / Total number of cases undergoing education on VTE prevention during the statistical cycle x 100% |  |  |  |  |  |  |  |  |  |  |  |  |
|  | III-29  Satisfaction of pregnant and postpartum women |  |  |  |  |  |  |  | = Sum of the actual scores of the received satisfaction questionnaire / Total scores of the satisfaction survey  questionnaire x 100% |  |  |  |  |  |  |  |  |  |  |  |  |
| Note: a, very important (5 points); b,important (4 points); c, general important (3 points); d,not very important (2 points); e,very unimportant (1 point);  f, very reasonable (5 points); g, reasonable (4 points); h, general reasonable (3 points); i,not very reasonable (2 points); j,very unreasonable (1 point);  k, very feasible (5 points); l, feasible (4 points); m, general feasible (3 points); n,not very feasible (2 points); o,very unfeasible (1 point). | | | | | | | | | | | | | | | | | | | | | |

**Part two Survey form of expert basic information**

Form completion instructions: This section contains a survey of the expert's general information and level of authority. Please fill in the information accurately or mark "√" for each item. Please do not miss any items!

| Gender |  | Age (years) |  | Years of working |  | Workplace |  |
| --- | --- | --- | --- | --- | --- | --- | --- |
| Degree of education |  | Title |  | Position |  | | |
| Job nature | Nursing management □ Clinical nursing □ Clinical medicine □ Clinical research □ Others □ | | | | | | |
| Familiarity with the research content | Very familiar □ More familiar □ General familiar □ Not very familiar □ Unfamiliar □ | | | | | | |
| Please mark "√" in boxes (large, medium, small) to indicate your judgment basis for the above indicators. | | | | | | | |
| Judgement basis | | Expert assessment | | | | | |
|  |  | Large | | Medium | | Small | |
| Theoretical analysis | |  | |  | |  | |
| Practical experience | |  | |  | |  | |
| Refer to domestic and foreign sources of information. | |  | |  | |  | |
| Intuitive judgment | |  | |  | |  | |
