# Supplementary material for: Construction of nursing-sensitive quality indicators for pregnancy-associated venous thromboembolism using the Delphi method
Source: BMC Nurs. 2026 Mar 7;25:358. doi: 10.1186/s12912-026-04517-y (PMC13081401; doi:10.1186/s12912-026-04517-y)
Supplement: Supplementary file 2 — Supplementary Material 2 [file 12912_2026_4517_MOESM2_ESM.docx]

**Supplementary Table 1**

| **The application method of each tertiary indicators in clinical settings** | |
| --- | --- |
| Tertiary indicators | Applicable method in clinical settings |
| Rate of implementation of nursing management system for VTE prevention | Nursing managers should establish VTE prevention nursing management system, forming a three-tier management structure comprised of ward nursing directors, team leaders, and charge nurses. Every obstetric nurse should be aware of and adhere to the management system, while nursing managers continuously refine it in clinical practice. |
| Rate of implementation of nursing standardization procedure for VTE prevention | Nursing managers should establish VTE prevention-related nursing procedures, and every obstetric nurse should be aware of these procedures and follow them in clinical practice.Nursing managers continuously refine the procedures in clinical practice. |
| Rate of implementation of assessment system for VTE risk | Nursing managers should establish VTE risk assessment system, and every obstetric nurse should be aware of it and apply it in clinical practice. Nursing managers continuously refine the procedures in clinical practice. |
| Ratio of obstetric specialist nurses for VTE prevention | Nursing managers should keep a record of the number of obstetric nurses who attend VTE specialist nurse training courses and obtain certificates, and regularly update the composition ratio of specialist nurses in obstetric VTE prevention. |
| Rate of completeness of VTE prevention equipment | Nursing managers should equip the department with VTE prevention devices such as graduated compression stockings, intermittent pneumatic compression devices, and plantar venous pumps. Regular inspections should be conducted to ensure that the equipment is in good working condition. |
| Rate of completeness of health education materials for VTE prevention | Nursing managers should equip the department with a variety of VTE prevention health education materials, such as educational manuals, videos, and posters. |
| Rate of qualified test of VTE theoretical knowledge | Nursing managers document the number of participants, timing, and scores for the VTE prevention theoretical assessments. They conduct root cause analysis and propose improvement measures based on their findings. |
| Rate of qualified test of operational skills for VTE prevention | Nursing managers conduct on-site evaluations of the VTE prevention nursing procedures performed by obstetric nurses and record the location, time, number of people, and scores of the evaluations. They also conduct cause analysis and improvement. |
| Rate of implementation of regular training for VTE team | Nursing managers should record the title, content, time, location, and participants of each training session and regularly review the training records. |
| Rate of VTE risk assessment | Nursing managers can refer to the 2015 edition of the Royal College of Obstetricians and Gynaecologists' risk assessment form and the 2020 edition of the Queensland Health Organization's assessment form, and tailor a VTE risk assessment form based on the actual situation in their hospital. |
| Rate of timely assessment of VTE risk | The charge nurse conduct VTE risk assessment for pregnant women upon admission, in case of pregnancy complications, immobilization, changes in the patient's condition, during labor and within 6 hours after delivery, and promptly document it in the nursing records including assessment content and time. Nursing managers regularly review nursing records to ensure that the charge nurse conducts timely and accurate risk assessments. |
| Rate of implementation of the first assessment of VTE bleeding risk | The charge nurse conducts a bleeding risk assessment before administering medication and promptly records the assessment time, content, etc. in the nursing record. Nursing managers regularly review nursing records to ensure that the charge nurse conducts bleeding risk assessments. |
| Rate of implementation of re-assessment of VTE bleeding risk | The charge nurse should reassess the bleeding risk of the pregnant and postpartum woman again after administering the medication and on the day of discharge, and promptly record the assessment time, content, etc. on the nursing record. Nursing managers regularly review nursing records to ensure that the charge nurse conducts bleeding risk re-assessments. |
| Rate of education on VTE prevention | The charge nurse should provide VTE prevention education to pregnant and postpartum women and promptly record the education time, content in the nursing records. Nursing managers regularly review nursing records to ensure that the charge nurse conducts VTE prevention education. |
| Rate of accurate education contents on VTE prevention | The charge nurse should provide VTE prevention education to pregnant and postpartum women, covering topics such as the risk factors for VTE, early symptoms and signs, the importance of preventive measures, potential side effects, and monitoring requirements. Nursing managers should conduct on-site observations to assess the charge nurse's ability to provide comprehensive and accurate VTE prevention education to pregnant women. |
| Rate of timely education on VTE prevention | The charge nurse is responsible for delivering VTE prevention health education to pregnant and postpartum women within 24 hours of admission, 24 hours post-operation, and 24 hours before discharge. Additionally, this information should be promptly documented in the nursing records, including specific details regarding the timing and content of the education. Nursing managers regularly review nursing records to determine whether the charge nurse has provided timely and accurate education. |
| Rate of administration of basic preventive measures | After assisting the pregnant and postpartum woman with implementing basic preventive measures (fluid intake, ankle pump exercises, early bed mobility), the charge nurse should promptly document these activities in the nursing record. Nursing managers regularly review nursing records. |
| Rate of administration of mechanical preventive measures | After assisting pregnant and postpartum women with the implementation of mechanical preventive measures (such as graduated compression stockings, intermittent pneumatic compression devices, and plantar venous pump), the charge nurse should promptly document these interventions in the nursing record. Nursing managers regularly review nursing records. |
| Rate of administration of drug prophylaxis measures | Low-molecular-weight heparin is the preferred anticoagulant for pregnant and postpartum women, and it is imperative that the charge nurse promptly documents the administration procedure in the nursing record. Nursing managers regularly review nursing records. |
| Rate of accurate nursing records on VTE prevention | Nursing managers regularly review the nursing records written by the charge nurses to check whether the care records related to VTE prevention are comprehensive and accurate, objectively record the quality control results, and find the reasons for improvement. |
| Rate of knowledge of VTE prevention | On the day of discharge, the responsible nurse conducted bedside interviews with pregnant and postpartum women to assess their understanding of VTE risk factors and prevention. The responses of all pregnant women who answered correctly were documented in a table, while any incorrect answers were also noted. Nursing managers routinely conduct an analysis of the awareness or lack of awareness of VTE prevention knowledge among pregnant women. |
| Incidence of hospital-acquired VTE | In the event of in-hospital VTE occurring in pregnant and postpartum women, the responsible nurse should promptly document clinical symptoms, onset timing, severity, treatment measures, and follow-up outcomes in the nursing records. Nursing administrators are required to routinely gather data on the incidence rate of in-hospital VTE and identify areas for improvement. |
| Incidence of complications related to VTE prevention measures | When pregnant and postpartum women experience complications related to VTE prevention measures, the charge nurse should promptly and comprehensively document the time of occurrence, severity, treatment process, and follow-up outcomes in the nursing records. Nursing administrators are required to routinely gather data on the incidence of complications related to VTE prevention measures and identify areas for improvement. |
| Incidence of adverse reactions related to anticoagulation | In the event of a pregnant or postpartum woman experiencing an adverse reaction related to anticoagulation (such as bleeding in various body parts, thrombocytopenia, abnormal liver function, and allergic reactions), it is imperative for the charge nurse to promptly and comprehensively document the event in the nursing record, including details on onset time, severity, treatment process, and follow-up outcomes |
